# Social Frailty in the COVID-19 Pandemic Era

**DOI:** 10.3389/fpsyt.2020.577113

**Published:** 2020-11-03

**Authors:** Madia Lozupone, Maddalena La Montagna, Ilaria Di Gioia, Rodolfo Sardone, Emanuela Resta, Antonio Daniele, Gianluigi Giannelli, Antonello Bellomo, Francesco Panza

**Affiliations:** ^1^Neurodegenerative Disease Unit, Department of Basic Medicine, Neuroscience, and Sense Organs, University of Bari Aldo Moro, Bari, Italy; ^2^Psychiatric Unit, Department of Clinical and Experimental Medicine, University of Foggia, Foggia, Italy; ^3^Population Health Unit - “Salus in Apulia Study” - National Institute of Gastroenterology “Saverio de Bellis,” Research Hospital, Bari, Italy; ^4^Department of Cardiac, Thoracic, and Vascular Science, Institute of Respiratory Disease, University of Bari Aldo Moro, Bari, Italy; ^5^Translational Medicine and Management of Health Systems, University of Foggia, Foggia, Italy; ^6^Institute of Neurology, Catholic University of Sacred Heart, Rome, Italy; ^7^Institute of Neurology, Fondazione Policlinico Universitario A. Gemelli Istituto di Ricovero e Cura a Carattere Scientifico, Rome, Italy

**Keywords:** SARS-CoV-2, coronavirus, social dysfunction, loneliness, immune system, biomarkers, Late-Life Depression (LLD), Multimorbidity (MM)

## Abstract

Special attention and efforts to protect from or reduce health-related outcomes of severe acute respiratory syndrome coronavirus 2 (SARS-CoV-2), the virus triggering coronavirus disease 2019 (COVID-19), should be applied in susceptible populations, including frail older people. In particular, the early death cases occurred primarily in older people with a frailty status, possibly due to a weaker immune system fostering faster progression of the viral infection. Frailty is an age-related multidimensional clinical condition defined as a non-specific state of vulnerability, identifying older people at increased risk of falls, institutionalization, hospitalization, disability, dementia, and death. Among frailty phenotypes, social frailty has been least studied. It considers the role of socioeconomic context as a vulnerability status later in life. COVID-19 does not affect all populations equally, and social inequalities contribute to drive the spread of infections. It was known that the perception of social isolation, e.g., loneliness, affects mental and physical health, but the implicated molecular mechanisms, also related to the immune system, and its associated cognitive and health-related sequelae, are poorly understood. The increasing psychological distress derived by prolonged exposure to stress due to the lockdown scenario, and the reduced sources of support, contributed to making heavy demands on personal resources, i.e., self-efficacy and interpersonal variables. So, perceived loneliness may be a factor associated with psychological distress and an outcome in itself. In the COVID-19 pandemic era, a correct assessment of social frailty may be essential in terms of the prevention of late-life neuropsychiatric disorders.

## Introduction

Data coming from epidemiological studies suggest an association between aging and the risk of developing life-threatening health problems and mortality related to the spread of severe acute respiratory syndrome coronavirus 2 (SARS-CoV-2), the virus implicated in coronavirus disease 2019 (COVID-19) (1). The hierarchical relationship and interlaced time courses of molecular, phenotypic, and functional aging domains have not yet been established in humans. Although justified and necessary, the COVID-19 lockdown will inevitably compromise mental health of susceptible age strata groups, especially frail older people. To understand the implications of a specific phenotype of the frailty construct named social frailty on mental health in the COVID-19 pandemic era, we need a good understanding of the time and metrics of aging, especially those indicating the continuum ranging from biological to phenotypic and functional aging (2).

The community healthcare professionals consider relevant the assessment of subtle biological, phenotypic and functional changes of mental health later in life, since the duration of the pandemic-related period of isolation remains uncertain. Neuropsychiatric consequences of brain damage or disease—i.e., mental disorders—can derive either from direct effects of infection on the central nervous system (CNS) or indirectly via the immune response or otherwise from medical therapy (3). Neurotropic and neuroinvasive effects of coronaviruses have been described in humans.

The present perspective article aims to explore the risk of social isolation and loneliness sequelae in older frail adults subjected to isolation measures during the ongoing COVID-19 pandemic, for both preventative and transmission-restricting purposes.

Social participation is an indicator of successful aging and important determinant of health-related outcomes, including mortality (4). According to a deficit accumulation approach to frailty phenotypes, social vulnerability can be measured as an index of social problems, or “deficits,” such that the more social deficits one has, the more vulnerability to adverse outcomes one has. It is important to note that mental health in the geriatric population requires to be integrate in the wider context of the health status of socially frail individuals, also in view of the possibility of a weaker immune system permitting faster progression of viral infection. Fulfillment of basic social needs is necessary to function adequately and experience social well-being, just as basic physical needs fulfillment is required to experience physical well-being. Social frailty could be considered as a lack of resources to fulfill one's basic social needs (5). A correct assessment of social frailty is needed to prevent late-life neuropsychiatric disorders precipitated by the COVID-19 pandemic.

## SARS-CoV-2 and Relationships With the Central Nervous System

The epidemiological criteria about COVID-19 spread, from China to 229 countries, soared out of control to reach a pandemic. Globally, at the end of June 2020, there were 9,277,214 confirmed cases of COVID-19 (6), encompassing a wide clinical spectrum extending from asymptomatic infection, mild upper respiratory tract illness, to severe viral pneumonia with respiratory failure. SARS-CoV-2 enters human host cells by means of a receptor little expressed in the brain, the angiotensin converting enzyme 2 receptor. Additionally, SARS-CoV-2 can pass to the brain by means of the cribriform plate nearby to the olfactory bulb, enabling the virus to reach and affect the CNS, contributing to neurological tissue damage and to COVID-19-related morbidity and mortality. In particular, the reported hyposmia suggests, as shown for SARS-CoV-2, a nasal infection pathway allowing a possible direct access to the CNS (3).

Findings from the study by Helms and colleagues suggested that the most common neurological features in the COVID-19 patients were non-focal: confusion, agitation, dysexecutive syndrome, and diffusely boosted reflexes (7). Preliminary data suggested that delirium, confusion, agitation, and altered consciousness, as well as symptoms of depression, anxiety, and insomnia were also common in patients with COVID-19 (8). The etiology of the neuropsychiatric consequences of COVID-19 infection is likely multifactorial, including direct effects of viral infection inside the brain, a procoagulant state inducing cerebrovascular disease, a physiological impairment in terms of hypoxia deriving from respiratory failure, the activation of the immunological cascade, and the indirect effects of medical interventions, social isolation, the psychological impact deriving from a novel, severe, potentially fatal illness, concerns about infecting other people, and social stigma.

The immune response in SARS-CoV-2 infection has a major importance and may induce a hyperinflammatory state similar to the hemophagocytic lymphohisticytosis, featuring a transitory condition of increased C-reactive protein, ferritin, and interleukin-6 levels. Some of the psychiatric multimorbidity aspects might be explained by the well-described interplay between inflammation and depression (9, 10).

## Social Inequalities and Mental Health in Older Age

Regardless of COVID-19 pandemic, social inequalities are reported in mental disorders: income inequality, low levels of expected social support and educational attainment affect social participation, contributing to social exclusion especially in older age (11). The positive mental effects of abundant, stable social interactions from a person with a wide social network, can alleviate stress deriving from negative life events, resulting in speed recovery from illness, and preserving psychological health (12).

**Figure 1:**
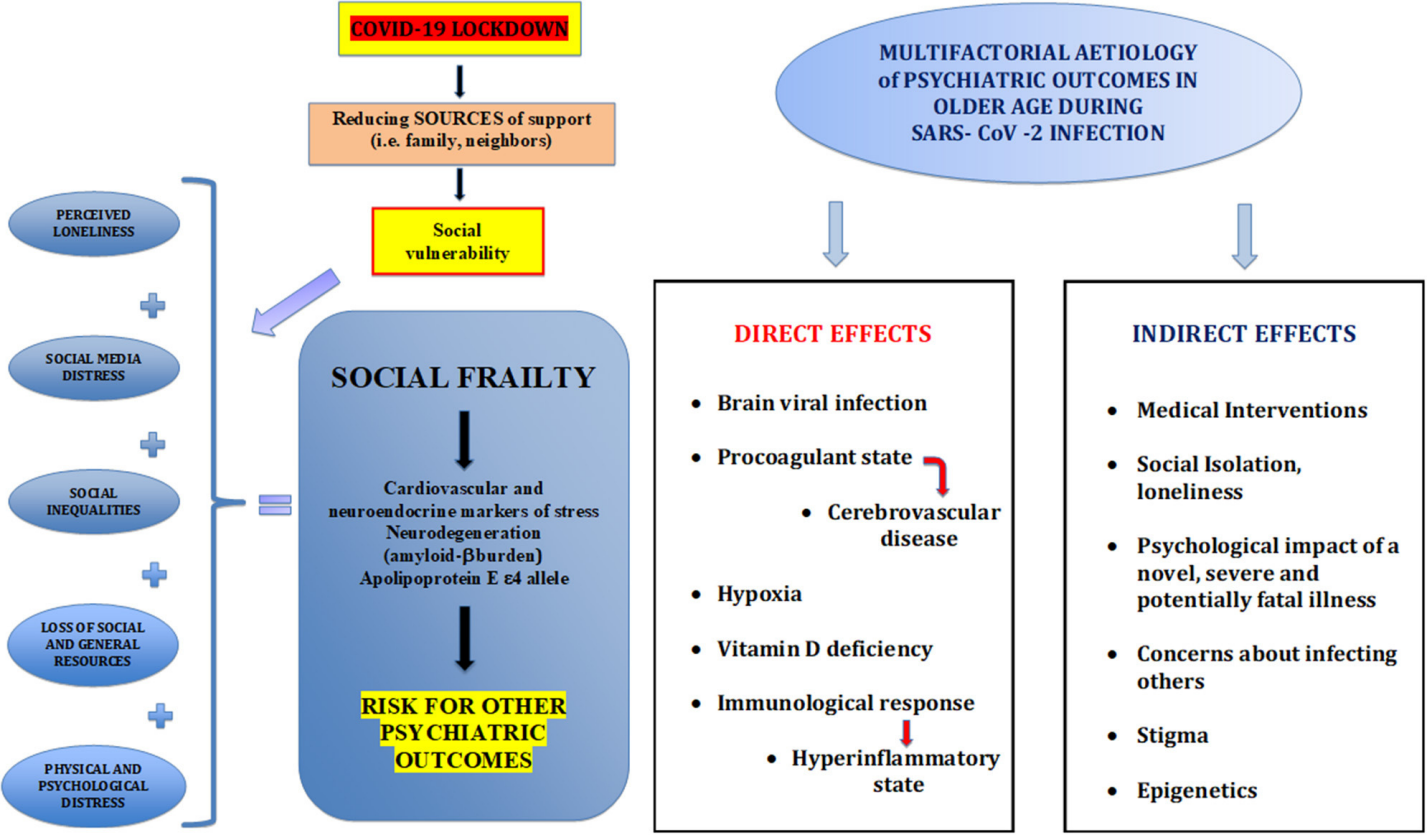
Relationship between coronavirus disease 2019 (COVID-19) lockdown and social resources and direct and indirect effects of severe acute respiratory syndrome coronavirus 2 (SARS-CoV-2), the virus causing COVID-19, on psychiatric outcomes in older age.

Everyday social environments rife with challenges represent an exposure to chronic stress whose proxy could be considered the socioeconomic status (SES) gradient. In the context of the current COVID-19 pandemic, there is a risk of increasing inequities in healthcare among vulnerable populations over the age of 65 and/or with multimorbidity, as potentially at-risk individuals (13). Furthermore, financial stress, transportation problems, and housing issues, as well as increased exposure to crime gained from a lower adolescent SES, may effectively compromise the benefits of a calm and mature personality on the stress response pathways, increasing the risk of dementia (14) (Figure 1).

## Loneliness, Social Isolation, and Late-Life Neuropsychiatric Disorders During the COVID-19 Pandemic: Mechanisms of Action

Several variables may explain why the current COVID-19 pandemic causes neuropsychiatric consequences especially in older age. The wider social impact of the pandemic and the legislative response, imposing physical distancing measures and quarantine, are among these reasons (15). The psychobiological etiological factors underlying anxiety and mood disorders arising at this social-environmental level should be better understood, as well as those at the genetic, molecular, or neural-circuitry level. An increasing body of research is focusing on the late-life depression–dementia interplay to probe the possible interaction between depression and aging mechanisms, including the influence of social determinants on epigenetic mechanisms (16).

Loneliness or emotional isolation is a subjective, undesirable experience, resulting from a cognitive mismatch between the quantity and quality of existing relationships and relationship standards. Loneliness has been viewed as a marker of psychosocial stress, resulting from depression, bereavement, and other social disconnection experiences. The downstream effects of loneliness on neural networks and systemic health is mediated by stress-related and inflammatory processes. In other words, great loneliness is linked to elevated cardiovascular and neuroendocrine markers of stress, impaired sleep, and proinflammatory physiological effects, that can cause neurodegeneration in the hippocampus and in other brain regions deputed to emotional regulation and cognition (17). Moreover, the amount of loneliness was associated to a greater brain amyloid-β (Aβ) protein burden after adjustment for demographic and clinical confounders, inversely Aβ-positive participants have a risk of 7.5 times higher to suffer from loneliness. These associations were stronger in apolipoprotein E ε4 allele carriers (18) (Figure 1).

Furthermore, physical activity and other healthy lifestyles worldwide were also affected by social distancing and quarantine restrictions (19). Home-isolation also tends to affect vitamin D levels by reducing the number of hours spent outdoors. There is evidence that vitamin D deficiency is linked to impaired immune function, potentially causing autoimmunity and increased risk of infections (20). Decreased levels of vitamin D might also determine a rise in mental health disorders (21).

It is interesting to note that, according to recent findings, psychosocial interventions (cognitive behavior therapy and multiple or combined interventions) are linked to an enhanced immune system function (proinflammatory cytokines or markers) and may therefore be useful for improving immune-related health (22, 23). It may be very important to study the mechanisms of psychosocial interventions and the link to beneficial effects on the immune system and health, particularly as related to COVID-19 infection (24). Epigenomics studies investigated molecular mechanisms by which loneliness exacerbates a wide range of neurodegenerative, psychiatric, and somatic diseases: Alzheimer's disease, psychiatric illness, immune dysfunction, and cancer gene sets seem to constitute essential targets for future investigations. The expression of pleiotropic genes at the time of death was found to be significantly enriched as a function of loneliness, experienced by a large sample of autopsied participants almost 5 years prior to death (25) (Figure 1).

## Psychosocial Determinants and COVID-19 Mortality

Older age is associated with greater mortality due to COVID-19. However, the vulnerability to physical comorbidities is not granted by physiological factors only, but also by psychosocial factors. Regardless of COVID-19 pandemic, it is known that different aspects of social relations are measured by social isolation and loneliness and both are slightly associated with different health outcomes and also mortality (social isolation to a greater degree than loneliness) (26). Currently, with the spread of COVID-19, social connectedness not necessarily is associated to higher mortality rate among older Italian adults (27). Inversely, variables associated with social isolation are found to be risk factors for an increased proportion of mortality in Italian patients aged 80 years and over. The conclusion could be that social relationships during a crisis impacting the frailest populations are a protective factor against increased mortality rates (27). Considering the lack of data on the acute effects of the illness, in terms of applicability to COVID-19, inferences must be drawn with care. Furthermore, no data on the post-illness phase have yet been described, although the higher COVID-19 mortality might be correlated with poorer psychiatric outcomes at a later date (8).

However, it should be made clear that physical frailty and social vulnerability (social frailty) are both entities clearly distinct, and that each contributes independently to mortality (28).

## Social Frailty

Frailty is a dynamic process and an intermediate state of aging, detrimental for health, involving a progressive reduction in physical, psychological and/or social functions. This condition has implication for public health linked to its multiple clinical and social consequences, as well as its dynamic nature also in terms of prevention (29). Physical frailty components such as a slower gait, exhibit significant reciprocal relationships with cognition, and may thus be a transitional step in the progression to late-life cognitive decline in some older adults. But the vulnerability of older adults does not appear to be completely explained by the biological perspective (physical or deficit accumulations approaches to frailty) (30). Different frailty phenotypes have been associated with a variety of socioeconomic, behavioral, and other clinical characteristics, including lower education, lower income, female gender, unmarried status, obesity, underweight, multimorbidity, and premorbid disabilities (31).

The biopsychosocial model of frailty may add important advantages in terms of both assessment and intervention targets. Influenced by a range of variables, it has been defined as a dynamic state affecting an individual who experiences injuries in one or more human function fields (physical, psychological, social), that increases the risk of adverse outcomes (32). Although different theories on social needs exist, social frailty can be defined as the continuum of progressive loss of social and general resources, activities, or abilities serving during the course of life to fulfill one or more basic social needs. The framework of social frailty takes into account the various types of social and general resources (or constraints), social behaviors and activities, and self-management abilities, utilized for accomplishing (or affecting) social needs (5). For example, the fulfillment of the need to love and to be loved, the need to feel that one is doing the “right” thing according to relevant others and oneself, and to be part of a group with shared values. Furthermore, the need to distinguish oneself from others by means of specific talents or assets.

Acute illness is less well-tolerated by frail patients, but the degree of disease severity and the degree of frailty are each important (33), particularly in the COVID-19 pandemic era. Most importantly, the type and severity of the presenting illness are important variables independently associated with the clinical outcome and ability to fully recover. There are other mediating factors: female sex (34), smoking (35), and social vulnerability (36) that also influence the risk related to frailty status. A recent study conducted in Japanese older adults evidenced an association between social frailty with both cognitive and physical functions (37). Further studies are needed to confirm the hypothesized association between social frailty and cognitive and physical function. Moreover, compared to physical frailty and cognitive impairment, social frailty is more strongly associated with the occurrence of depressive symptoms among community-dwelling older adults after 4 years of follow-up (38). In other words, a greater incidence of neuropsychiatric disorders, directly proportional to the social frailty status, may be expected as late consequences of the COVID-19 pandemic.

## Social Frailty Assessment Tools

Deficit accumulation model of frailty can be understood to occur at many levels, from the (sub-)cellular level to tissues, organisms/complex systems and societies. Deficits can also accumulate at the tissue level and at the level of complex systems, i.e., in individual people or animals, which are effectively complex systems. Of particular relevance to the present discussion, deficits can also accumulate at levels higher than individuals, e.g., at the social level, pertaining to social environments and circumstances, and these are the clinical epiphenomena that we need to measure. More complex tools to evaluate social frailty basis beyond symptom scales/health checklists are needed (genetics, laboratory-based biomarkers, neuroimaging, etc), because social frailty could be considered a complex clinical phenotype.

Mental healthcare clinicians face substantial time challenges, including limited time available for evaluation for therapy, which does not yet include standardized assessments of social isolation and loneliness. The question is: what are we measuring to make interventions? Loneliness, social isolation, social relationships or all three? Social frailty is usually evaluated by single questions or items deriving from functional and depressive symptom scales or health checklists. But each of these instruments measures only partial aspects deriving from structural or functional aspects of social relationships, and is based on subjective responses (39).

Social frailty has been operazionalized with single questions or items from functional and depressive symptom scales or health checklists (40). A shared opinion is that there is heterogeneity in the definition of social frailty in different studies, and there is a request of homogeneity and simplification in the instruments of this assessment. Social contact, participation, depression, and loneliness characterize different scales of assessment of different social frailty models (41–44). Also, well-known instruments for assessing frailty in community-dwelling older people such as the Tilburg Frailty Indicator (45), aim to assess physical, psychological, and social frailty and their health-related outcomes (4). The Social Vulnerability Index, as a method of quantification of social vulnerability, predicted long-term mortality in different population-based settings and could have a role in this context of COVID-19 pandemic (46).

Recently, the Social Dysfunction Rating Scale (SDRS) was validated with a proposed cut-off for detecting social frailty in older age, for the purposes of considering possible interventions to maintain healthy aging (47). SDRS items are a mixture of subjective and objective evaluations considering both the rater's opinion and the subject's own self-evaluation. The scale includes important elements of functioning such as personal satisfaction and self-fulfillment and takes into account social role performance only peripherally (48). For example, among the items evaluating self-system, questions about self-concept, goallessness, meaning in life, self-health concerns are asked to the subjects; the investigation of interpersonal system implies to ask about emotional withdrawal, hostility, anxiety etc. Finally, questions about performances system consist in investigating lack of satisfying relationships with significant persons, express need for social contact or friends, lack of satisfaction from work, expressed need for more leisure activities, financial insecurities, etc.

The SDRS could be a valid instrument to capture size (isolation) and quality (loneliness, neuroticism) of social adjustment in older age. The perception of social dysfunction was not associated with material deprivation, and this adds another stratum of complexity in the assessment of health status in older age. Factorial analysis of SDRS's twenty-one items was performed (47) and five factors were identified for the 21-item SDRS, according to their loads in the analysis: social isolation; loneliness; feelings of contribution/ uselessness; lack of leisure activities; anxiety for the health. Furthermore, SDRS was correlated to cognitive (apathy, Mini-Mental State Examination, and Frontal Assessment Battery) and psychiatric outcomes.

**Figure 2:**
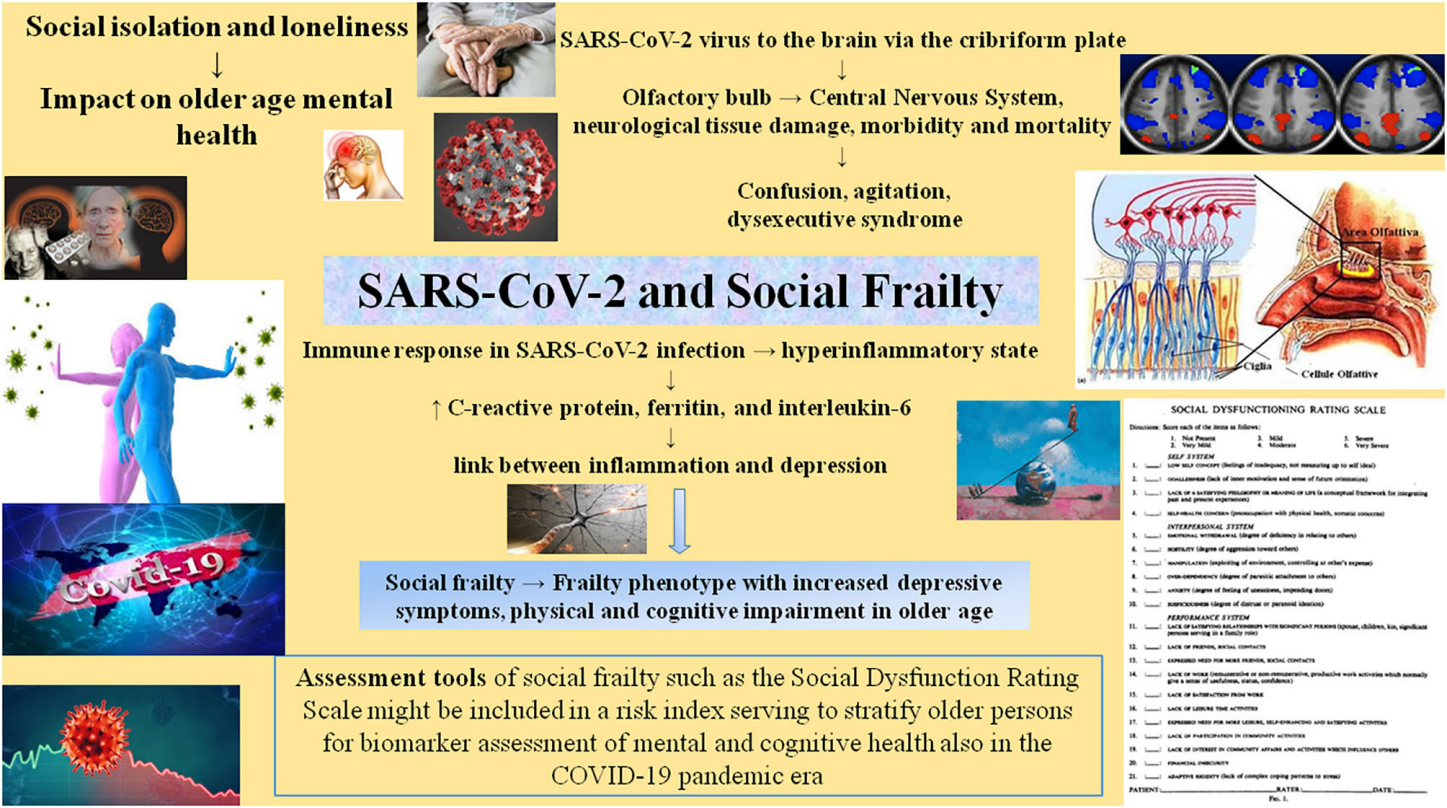
The influence of severe acute respiratory syndrome coronavirus 2 (SARS-CoV-2) on mental health in older age can impinge on social frailty, a particular frailty phenotype that has not yet been assigned a full, universally recognized definition. The mechanisms by which social isolation and loneliness affect mental health are now under study, also in terms of how best to assess them.

These concepts and findings may help us to develop low cost methods for screening older persons for mental health outcomes, and selecting participants for prevention interventions (48). In the present perspective article, we underline the importance of early detection and interventions on dysfunctional aspects of social functions. Social functioning and the SDRS might be included in a risk index helping to stratify older persons for biomarker assessment of mental and cognitive health (i.e., late-life depression or dementia) also in the COVID-19 pandemic era (49, 50) (Figure 2).

## Discussion

Loneliness could be tackled with various interventions (51), broadly divided into two categories, that is, social interventions and technological interventions. Social interventions applied to reduce loneliness include befriending, residential and school-based camps, reminiscence therapy, animal interventions, gardening, physical activity and technology (52). However, in older people, loneliness can create serious problems that could not be alleviated with the social support only (53). Therefore, particular in older age, other types of interventions are required such as technological interventions (i.e., digital applications, online social networks and social robots) to enhance emotional support and social interaction (54).

The media, such as television and radio, have become ever more important during the period of social isolation due to COVID-19 lockdown. However, in this climate, the level of distress and anxiety can height, because of news coverage. Internet, electronic communication and, first and foremost, social media, had offered an extraordinary increase in connectivity between people and societies, playing a principal role in forcing the spread of bad news and in deepening the impact of the worldwide major problems on mental health, considered an outcome. Thanks to the help of video calls, older people stay connected during the current crisis. In this way, they widen their social circle and increase the frequency of contact with existing contacts. However, based on a recent review, evidence of the efficacy of video call interventions in reducing older adults' loneliness, is currently very uncertain (55), as was the evidence of their effectiveness as a means of evaluating outcomes of symptoms of depression (55). More rigorous methods and larger samples of participants are required in terms of future standpoint for this area of research.

The future perspective of old age psychiatry in COVID-19 pandemic is to cope with the framework of negative moods, stress and socially mediated traumatic experiences and adverse developments deriving from social epidemiology (56). Social determinants may positively modulate the effects of epigenetic factors on neuropsychiatric disorders in older age also *via* the modulation of immune system. In the future, social incentive exposure—which relies on patient social and physical activation—could be a potential mechanism of treatment for different psychiatric disorders, including late-life depression (57). Moreover, social isolation and loneliness is a potentially modifiable risk factor for later psychiatric multimorbidity that may offer an opportunity to enhance psychiatric care in new ways by addressing the underlying causes, and building coalitions to increase engagement and support by others outside the healthcare system (50).

## Data Availability Statement

The original contributions generated for this study are included in the article/supplementary material, further inquiries can be directed to the corresponding author/s.

## Author Contributions

ML: conceptualization. ML and FP: manuscript writing and supervision. MLM and ID: drawn the figures. RS, AD, ER, GG, and MM AB: contributed to the bibliographic search, review, and editing.

## Conflict of Interest

The authors declare that the research was conducted in the absence of any commercial or financial relationships that could be construed as a potential conflict of interest.
